# On Yaglom’s Law for the Interplanetary Proton Density and Temperature Fluctuations in Solar Wind Turbulence

**DOI:** 10.3390/e22121419

**Published:** 2020-12-15

**Authors:** Giuseppe Consolini, Tommaso Alberti, Vincenzo Carbone

**Affiliations:** 1 INAF-Istituto di Astrofisica e Planetologia Spaziali, Via del Fosso del Cavaliere, 100, 00133 Roma, Italy; tommaso.alberti@inaf.it; 2 Dipartimento di Fisica, Università della Calabria, Ponte P. Bucci, 87036 Rende, Italy; vincenzo.carbone@fis.unical.it

**Keywords:** solar wind, Yaglom’s law, magnetohydrodynamics, turbulence, space plasma

## Abstract

In the past decades, there has been an increasing literature on the presence of an inertial energy cascade in interplanetary space plasma, being interpreted as the signature of Magnetohydrodynamic turbulence (MHD) for both fields and passive scalars. Here, we investigate the passive scalar nature of the solar wind proton density and temperature by looking for scaling features in the mixed-scalar third-order structure functions using measurements on-board the Ulysses spacecraft during two different periods, i.e., an equatorial slow solar wind and a high-latitude fast solar wind, respectively. We find a linear scaling of the mixed third-order structure function as predicted by Yaglom’s law for passive scalars in the case of slow solar wind, while the results for fast solar wind suggest that the mixed fourth-order structure function displays a linear scaling. A simple empirical explanation of the observed difference is proposed and discussed.

## 1. Introduction

The interplanetary medium is permeated by a supersonic, super-Alfvénic magnetized plasma flow, the solar wind, whose origin is in the outer solar atmosphere. It is characterized by large-amplitude, scale-invariant, turbulent, and intermittent fluctuations over a wide range of scales. At large scales (down to the ion gyroscale), solar wind fluctuations are generally described within the framework of Magnetohydrodynamic (MHD) turbulence [1], whose scaling behavior follows a Kolmogorov-like $k^{-5/3}$ law [1,2,3]. The presence of intermittency in solar wind field fluctuations leads to a steeper spectrum than the MHD inertial range scaling predicted by Kraichnan [4]. Indeed, especially by means of the Ulysses mission, the intermittent nature of the interplanetary medium turbulence was widely investigated both on the experimental/observational side and the theoretical one [1]. The direct evidence of an inertial energy cascade, as predicted by MHD Alfvénic turbulence theory, has been only recently found and reported in a series of works on the linear dependence of the third-order structure function of Elsässer variables for high-latitude solar wind [5,6]. All these past and recent results make the solar wind a natural laboratory to study hydromagnetic turbulence and related phenomena.

In the framework of turbulent media, the advection and diffusion of scalar and vector fields have been some of the main issues in the last few decades [7,8,9,10]. Examples of passive scalars in fluids are diffusive contaminants, dye blobs, heat, temperature, etc.

The behavior of a scalar field ϕ(r,t) passively advected by a turbulent velocity field u(r,t) is governed by the advection-diffusion partial differential equation,

(1) $$\partial_{t}\phi \left(r,t\right)-u\left(r,t\right)\cdot \nabla \phi \left(r,t\right)=\kappa \Delta \phi \left(r,t\right)+f\left(r,t\right)$$

where u is the turbulent velocity field, κ is the molecular diffusivity coefficient, $\Delta =\nabla^{2}$, and *f* is a forcing term necessary to attain a stationary state.

Converse to the case of zero molecular diffusion, which implies a rigid advection of the scalar field, a small amount of diffusivity gives rise to a different behavior, similar to that of flow turbulence, which results in the formation of an inertial range where scalar fluctuations cascade from large to small scales at a constant scale-independent rate $\epsilon_{\phi }$. Moving from a description analogous to that of Kolmogorov’s theory of turbulence, Obukhov and Corrsin [11,12] predicted the following scaling of the scalar second-order structure function $S_{2}\left(r\right)$,

(2) $$S_{2}\left(r\right)=\langle \delta_{r}\phi^{2}\rangle ∼\epsilon_{\theta }\epsilon^{-1/3}r^{-2/3},$$

where $\delta_{r}\phi =\phi \left(x+r\hat{e}\right)-\phi \left(x\right)$, $\epsilon_{\theta }$ is the dissipation rate of the scalar variance, and ϵ is the energy dissipation rate of the turbulent velocity field. Consequently, the expected expression of the spectral density of the passive scalar in the inertial range, where the diffusive effects are negligible, is the usual 5/3-law, i.e.,

(3) $$S_{\phi }\left(k\right)∼k^{-\frac{5}{3}}.$$

Today, it is known that in such turbulent flows, anomalous and super diffusion is present (see, e.g., [3,13,14]). This physical phenomenon influences the exponent of Equation (3). However, as for interplanetary flows, the Reynolds number is extremely high, the result (3) may be considered to be the correct one. Moreover, as in the case of Kolmogorov’s theory of turbulence, the constancy of the scalar flux in the inertial range results in an exact, model-independent relation, namely Yaglom’s law [15], for the scalar-mixed moment. This relation is the scalar turbulence counterpart of the well-known 4/5 Kolmogorov’s law and assumes the following form,

(4) $$\langle \left[\delta_{r}u\cdot \hat{r}\right]{\left[\delta_{r}\phi \right]}^{2}\rangle =-\frac{4}{3}\epsilon_{\phi }r,$$

which is strictly valid within the inertial range. All the previous considerations are clearly valid in the case of isotropic and homogeneous 3D turbulence. For instance, in the case of 2D isotropic turbulence, the linear scaling predicted by Yaglom’s law, Equation (4), assumes a slightly different form where the pre-factor is −2 instead of −4/3.

In a preliminary work [16], one of us investigated the scaling and intermittent features of solar wind proton temperature, showing how the behavior of such a plasma parameter is analogous to that of a turbulent advected passive scalar. In detail, it was found that the scaling exponents of structure functions of proton temperature are very well in agreement with experimental results relative to the scaling features of temperature fluctuations in neutral fluid turbulence [17]. Furthermore, in a recent work, Consolini et al. [18] showed that also the electron density fluctuations in turbulent polar ionospheric plasma display intermittent features that are consistent with those of advected passive scalar quantities.

Here, we investigate the presence of an inertial range by verifying the presence of a linear dependence of the third-order scalar-mixed moment for the proton density and temperature, as predicted by Yaglom’s law, in two long-lasting periods of different (fast and slow) solar wind conditions, observed by the Ulysses spacecraft. In the case of proton temperature, this work should be considered as a preliminary analysis of Yaglom’s law due to the impossibility of investigating the effect of proton temperature anisotropy on the scaling features of the third-order scalar-mixed moment using Ulysses measurements.

## 2. Data Description

The presence of a power law with exponent 4/3 (Yaglom’s law) for solar wind proton density and temperature is investigated by using two periods of different solar wind conditions:

- (i) a period of six months from 01 January 1996 to 30 June 1996 (labeled as #1), when the Ulysses satellite was out of the solar equatorial plane (HGI Lat $\in (33^{\circ },53^{\circ })$) at a distance of ∼3.6 A.U., still observing high-speed solar wind (〈u〉=[750±20] km/s);
- (ii) an already investigated long-standing period of nearly constant slow solar wind conditions (〈u〉=[370±20] km/s) from 01 October 1997 to 31 March 1998 (labeled as #2), when the Ulysses satellite was on the heliospheric equatorial plane (HGI Lat $\in (-6^{\circ },4^{\circ })$) at a distance of ∼5 A.U. (see, e.g., [19,20]).

The two selected time intervals correspond to periods of small-to-moderate solar activity being localized around the solar minimum. Here, we use the solar wind velocity u, the proton density *n*, and temperature *T* measured by the SWOOPS (Solar Wind Observations Over the Poles of the Sun) instrument (PI D. McComas, Southwest Research Institute, USA) of the Ulysses satellite. Data came from the NASA-CDAWeb (website address http//cdaweb.gsfc.nasa.gov) and refer to UY_M0_BAI dataset. The typical time resolution of measurements is ∼4–8 min. Here, we downsampled data measurements to 8 min and interpolated missing points using a linear interpolation. The total number of available points for the two considered time intervals is $N_{p}\geq$ 30,000, which is a sufficient number to provide a correct estimate of the third-order mixed moment of Equation (4) with a precision of some percent (5÷10%) [21]. This point has been checked by means of a Monte Carlo numerical simulation (i.e., perturbing data by summing a Gaussian noise having a standard deviation equal to the expected mean data error).

The used dataset of plasma experiment on-board the Ulysses satellites provides two different estimates $T_{Small}$ and $T_{Large}$ of the proton temperature. These two estimates can be considered as an upper and lower estimate of the real proton temperature; $T_{Small}\leq T\leq T_{Large}$. As already observed in [20] for the low-latitude period, the two temperatures display the same statistical behavior also in the case of the high-latitude time interval considered here. Thus, we will consider the mean value between the two as a reliable estimate of the true proton temperature *T*. Furthermore, the average proton temperature is $\langle T\rangle =\left[110\pm 20\right]\cdot 10^{3}$ K, and $\langle T\rangle =\left[30\pm 20\right]\cdot 10^{3}$ K for the high-latitude fast solar wind period and the low-latitude slow solar wind one, respectively. These average proton temperatures agree with previous observations of fast and slow solar wind conditions [1,22].

**Figure 1:**
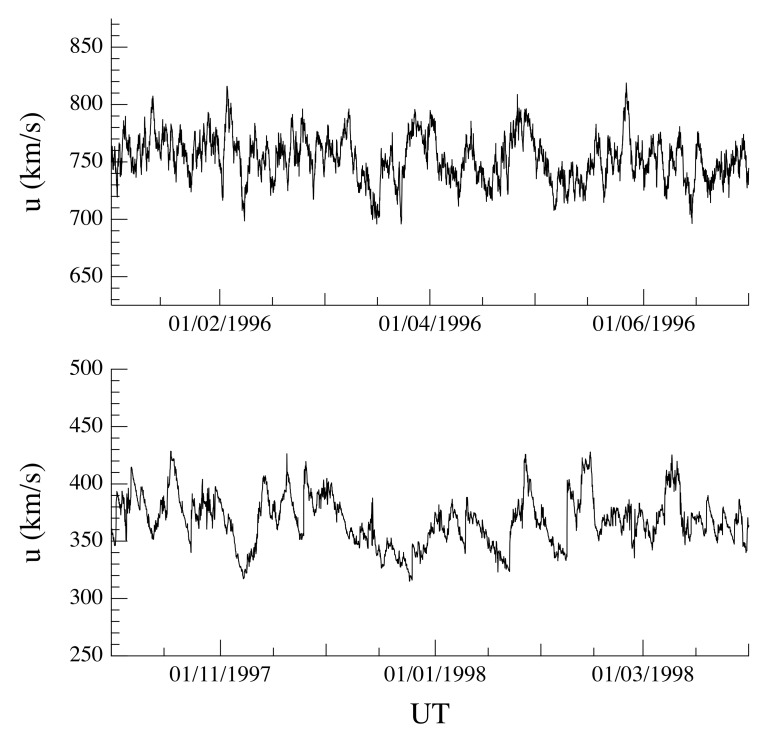
The solar wind flow velocity *u* for the two selected time intervals at hourly resolution (for visual purposes) as a function of the universal time (UT).

**Figure 2:**
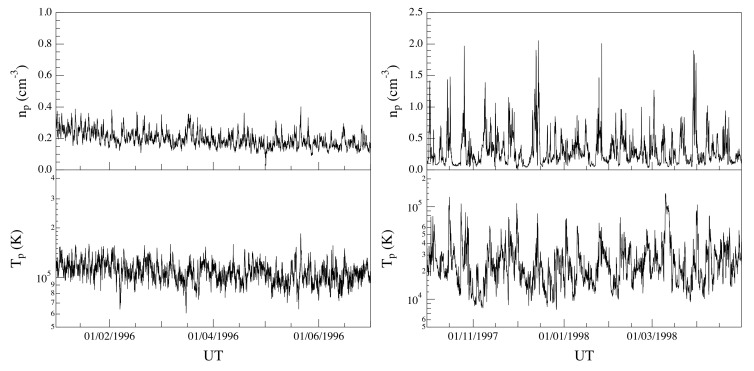
The solar wind proton density *n* and temperature *T* for the two selected time intervals at hourly resolution (for visual purposes) as a function of the universal time (UT).

**Figure 3:**
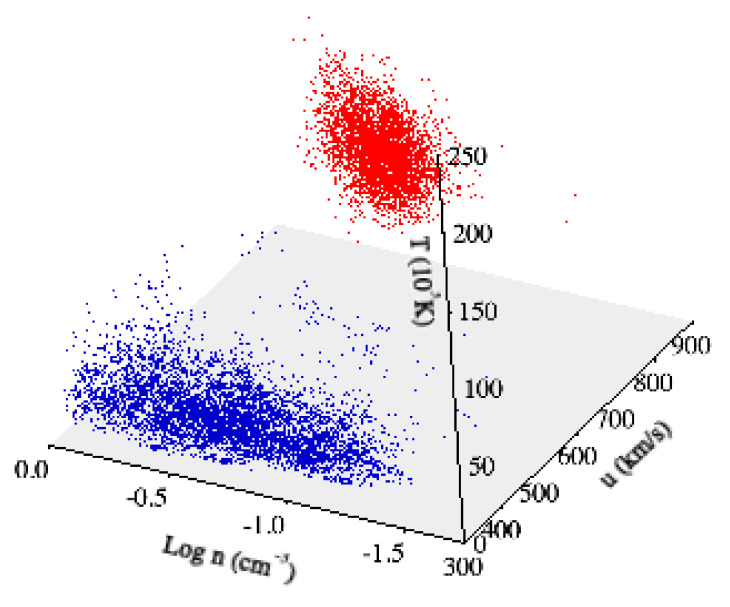
Scatter-plot of the two datasets in the 3D space (u,n,T). Blue and red dots refer to slow and fast solar wind, respectively.

Figure 1 and Figure 2 show the solar wind flow velocity *u*, the proton density *n*, and the temperature *T* for the two selected time intervals, respectively, at hourly resolution (for visual purposes). We note that for all the considered periods, the solar wind conditions can be considered quasi-stationary, although with different average values (∼750 km/s and ∼350 km/s for both the fast and the slow solar wind streams, respectively). This difference, in terms of solar wind conditions, can be also easily highlighted by means of the scatter plot of the data associated with the two selected periods in the 3D phase-space (u,n,T) (see Figure 3). Indeed, the nature of the 3D phase-space is clearly different between the two periods: the slow solar wind shows an elongated phase-space geometry, being a reflection of the larger variability of density fluctuations, while the fast solar wind is characterized by a localized geometry, being related to the low variability (in terms of fluctuations) of both density and temperature. Finally, the non-superposition of both periods provides the evidence of different dynamical states for the solar wind.

## 3. Methods

To investigate the occurrence of scaling features in the third-order mixed structure function and to compare this with the predictions of Yaglom’s law for passive scalar quantities, we here adopt a novel technique based on the use of the Empirical Mode Decomposition (EMD) [23] and named EMD-based Dominant Amplitude Multifractal Formalism (EMD-DAMF) [24].

### 3.1. The Empirical Mode Decomposition

The Empirical Mode Decomposition (EMD) is a data analysis method that has been developed to carry out a finite set of embedded modes from a given time series x(t) by using an iterative process known as the sifting process. Given a time series x(t), through the EMD, we can write:

(5) $$x\left(t\right)=\sum_{k=1}^{N}c_{k}\left(t\right)+r\left(t\right)$$

where r(t) is the residue of the decomposition [23]. For more details about the sifting algorithm, the reader is referred to [23,25].

The EMD provides non-stationary oscillating components whose amplitude-frequency modulation can be investigated by means of the Hilbert Spectral Analysis (HSA), e.g., [23,26]. Indeed, via the Hilbert Transform (HT), we can write each empirical mode $c_{k}\left(t\right)$ as modulated both in amplitude and in phase:

(6) $$c_{k}\left(t\right)=ℜ\left\{a_{k}\left(t\right)e^{\left[i\int_{0}^{t}\phi_{k}\left(t^{\prime }\right)dt^{\prime }\right]}\right\}$$

$a_{k}\left(t\right)$ and $\phi_{k}\left(t\right)$ being the instantaneous amplitude and phase of the *k*-th empirical mode, respectively, and *ℜ* is the real part. In this way, we are able to investigate non-stationary features of time series, $\phi_{k}\left(t\right)$ being a function of time, e.g., [23,27].

### 3.2. The EMD-Based Dominant Amplitude Multifractal Formalism

The EMD-DAMF [24] has been proposed to investigate singularities and the (multi)fractal behavior of time series in a similar way to the Wavelet Transform Modulus Maxima (WTMM). Indeed, it consists of the following steps:

1. derive the instantaneous amplitude $a_{k}\left(t\right)$ and mean timescale $\tau_{k}=\frac{1}{2\pi }{\langle \frac{d\phi_{k}\left(t\right)}{dt}\rangle }_{t}^{-1}$ of each empirical mode;
2. evaluate the dominant amplitude coefficients $u_{l,m}$ over a time support $I_{l,m}$ around the *l*-th local maximum:
  (7) $$u_{l,m}≐\underset{m^{\prime }\leq m}{\sup}\left\{\max\left\{|a_{m^{\prime }}\left(t\in I_{l,m}\right)|\right\}\right\}$$
  with $l=1,\cdots ,N_{m}$, $N_{m}$ being the number of local maxima of $a_{m}\left(t\right)$, and m=1,⋯,N;
3. evaluate the *q*-th-order structure function $S_{q}\left(\tau_{k}\right)$:
  (8) $$S_{q}\left(\tau_{k}\right)=\frac{1}{N_{m}}\sum_{l=1}^{N_{m}}{\left\{u_{l,m}\right\}}^{q};$$
4. derive the scaling exponent ζ(q) as:
  (9) $$S_{q}\left(\tau_{k}\right)∼\tau_{k}^{\zeta (q)};$$
5. use the Legendre transform to evaluate singularities α and their spectrum f(α)
  (10) $$\alpha =\frac{d\zeta (q)}{dq}\;\&\;f\left(\alpha \right)=\alpha q-\zeta \left(q\right).$$

In this way, we can exploit local features of empirical modes to investigate the high-order statistics of increments at different timescales without a priori defining the timescales, but by using local extrema to compute the differences/increments between two points. Moreover, the limited numbers of timescales, due to the small number of empirical modes derived via the EMD, allow visually investigating the scaling laws and better characterizing the scaling exponents with respect to the classical structure function analysis [28].

## 4. Analysis and Results

**Figure 4:**
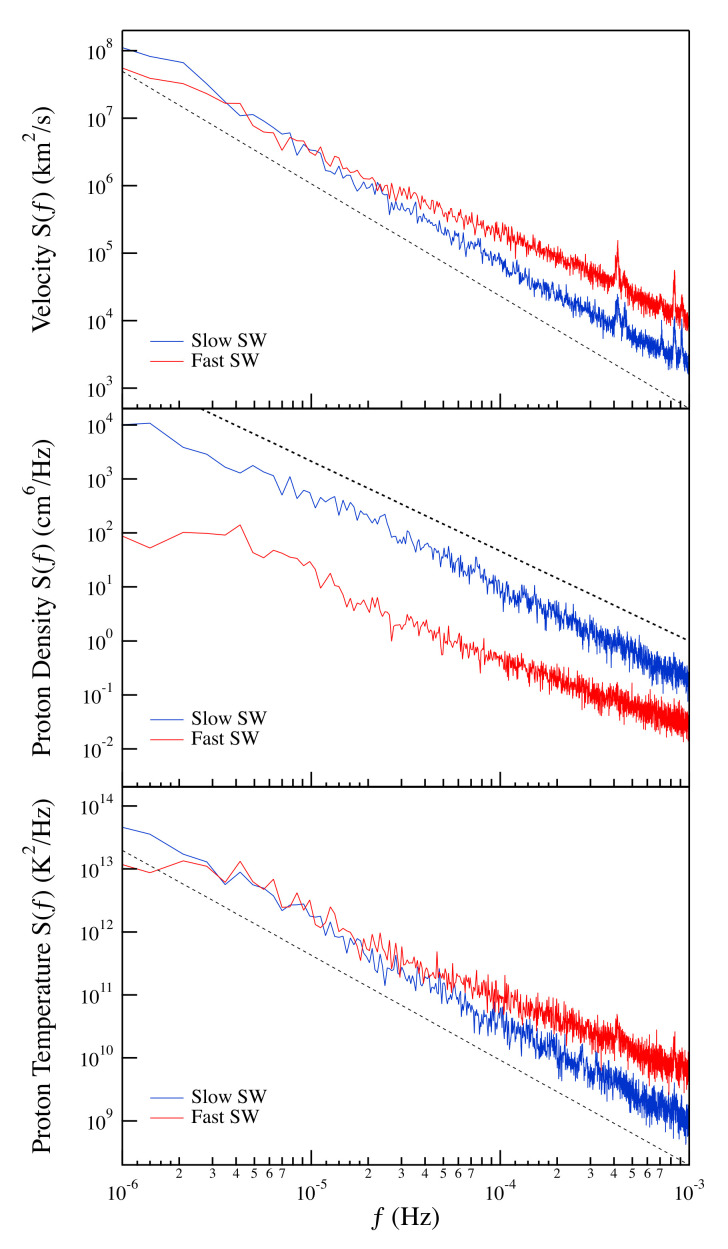
Power spectral density of the solar wind (SW) velocity, the proton density, and the proton temperature for the two selected periods of fast and slow solar wind conditions. The dashed line refers to the −5/3 power-law as predicted from KOC theory.

As a first step of our analysis, we investigate the spectral features of the selected quantities (*u*, *n*, and *T*) for the two selected time intervals as already done in a previous work [16]. Figure 4 shows the spectral features of the quantities under investigation for the two selected periods.

The observed spectral features shows the occurrence of clear frequency domains characterized by a power-law behavior of the spectral density, $S\left(f\right)∼f^{-\beta }$. In particular, in the case of slow solar wind, the observed spectral exponent for all the quantities under investigation is very well in agreement with the expected behavior from the KOC theory [11,12], which predicts an exponent β=5/3. Differently, the spectral density for the fast solar wind is less steep, showing a spectral exponent β∼3/2.

**Figure 5:**
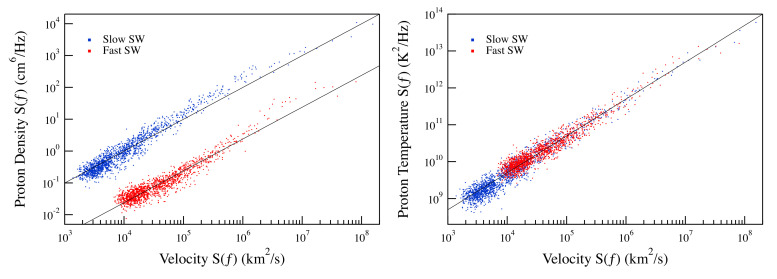
Behavior of the proton density and temperature spectra as a function of the velocity spectra for the fast and solar wind periods. The solid lines refer to a linear dependence.

As shown in Figure 5, in spite of the different spectral features between the slow and fast solar wind periods, we observe a linear dependence of the proton density and temperature spectra with respect to the velocity one ($S_{n},S_{T}∼S_{u}$). This linear dependence is very good in the case of the proton temperature extending over more than three orders of magnitude. Conversely, some slight discrepancies are observed in the case of the proton density especially in the case of the slow solar wind period. The observed small discrepancy could be due to an anomalous scaling, which in the case of passive quantities is more pronounced and could imply a different intermittency correction to the spectral exponent [17,29]. The quasi-linear dependence of the spectra of the proton density and temperature on the velocity spectra support the hypothesis that these quantities could behave as scalars passively advected by the solar wind.

To check this point, we compute the mixed third-order structure function reported in Equation (4) using the EMD-DAMF method described in the previous Section 3.2. For correctly evaluating the mixed third-order structure function, we use the velocity radial component, which is the one parallel to the solar wind direction. Furthermore, because our estimation of the increments of the investigated quantity is made with respect to time scale τ, according to previous works [5,30], these local time measurements are then transformed into spatial measurements by using the Taylor hypothesis, that is $r=-u_{0}\tau$ (note the reversed sign), where $u_{0}$ is the mean radial velocity over the selected period. Consequently, Yaglom’s law, expressed in terms of the time scale τ, is:

(11) $$\langle \left[\delta_{\tau }u_{R}\right]{\left[\delta_{\tau }\phi \right]}^{2}\rangle =\frac{4}{3}\epsilon_{\phi }u_{0}\tau ,$$

where $\delta_{\tau }y=y\left(t+\tau \right)-y\left(t\right)$. The time scale τ is set to be half of the characteristic periodicity $T_{IMF}$ of each IMF obtained by the EMD decomposition, i.e., $\tau =T_{IMF}/2$. Here, we investigate the presence of Yaglom’s law over a time-delay interval from ∼12 min up to ∼9500 min, which approximately corresponds to a spatial interval of $\left[4\times 10^{-3};3\right]$ A.U. and $\left[2\times 10^{-3};1.5\right]$ A.U., for fast and slow solar wind conditions, respectively. We remark that, being that the passive scalar dynamics is governed by an advection-diffusion equation, namely Equation (1), these dissipation rates are mainly related to the role of the diffusion of passive quantities across the inertial range.

**Figure 6:**
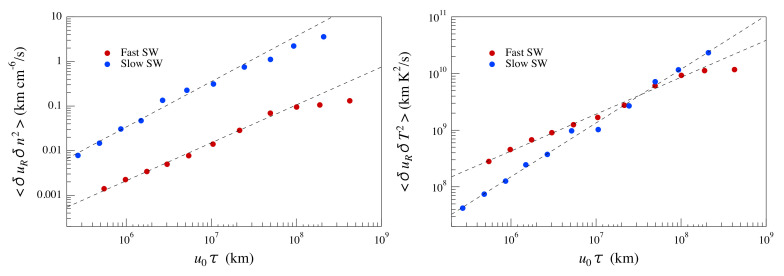
The mixed third-order structure functions for the proton density (**left** panel) and the proton temperature (**right** panel) as a function of $u_{0}\tau$ for the two selected periods of fast and slow solar wind. Dashed lines correspond to power-law fits whose exponents are reported in Table 1.

Figure 6 shows the behavior of the mixed third-order structure functions for the proton density and temperature as a function of $u_{0}\tau$ for the two periods of fast and slow solar wind. To investigate the presence of a linear scaling as predicted by Yaglom’s law for passive scalar quantities, we fit the observed trends of the mixed third-order structure functions using a power law fit, $\langle \left[\delta_{\tau }u_{R}\right]{\left[\delta_{\tau }\phi \right]}^{2}\rangle =A{\left(u_{0}\tau \right)}^{\alpha }$. The details of the fit results are reported in Table 1.

By looking at the results reported in Table 1, one can immediately realize that the mixed third-order structure functions of proton density and temperature for slow solar wind practically follow a linear scaling as predicted by Yaglom’s law for passive scalar quantities over a wide range of scales. In such a case, we can evaluate the corresponding dissipation rates of the density and temperature variance $\epsilon_{\phi }$, under the assumption of a 3D homogeneous and isotropic turbulent regime, i.e., assuming the four-thirds law. In such a case, we get $\epsilon_{n}∼7.5\cdot 10^{-9}$ cm${}^{-6}$/s and $\epsilon_{T}∼120$ K${}^{2}$/s for the proton density and temperature, respectively.

Conversely, in the case of fast solar wind, we found a clear discrepancy from the expected Yaglom’s law. Indeed, although there is a clear scaling over a wide range of scales, the observed scaling exponents significantly deviate from α=1. This could be due to the occurrence of a correction to the scaling (e.g., an effect due to a scale dependence of the energy transfer rate), whose origin it is not clear at the present stage.

## 5. Discussion and Conclusions

In this work, we study the passive scalar character of proton density and temperature in fast and slow solar wind by investigating the occurrence of Yaglom’s law for the third scalar-mixed moment. Our analysis is performed by applying the recent technique of EMD-DAMF [24], which is based on the EMD introduced by [23]. This is one of the first applications of such a method in the framework of space physics to investigate passive scalar features.

Our results suggest that in agreement with the Yaglom law’s prediction, the passive character of the two investigated solar wind quantities is verified in the case of the slow solar wind by the linear scaling of the third-order scalar-mixed structure functions of the passive quantities, which extends over a wide range of scales. Indeed, the linear scaling of the third-order scalar mixed moment is the signature of the presence of a well-defined inertial range for the proton density and temperature turbulent fluctuations. We note that the observed inertial range for the proton density spans over a number of decades, which is comparable to what was previously observed by Sorriso-Valvo et al. [5].

On the other hand, in the case of fast solar wind, the situation is less clear. Indeed, in this case, although we find scaling features of the proton density and temperature third-order scalar-mixed structure functions, the observed scaling properties do not agree with the predictions of Yaglom’s law, which is usually recovered in ordinary fluid flows. Although we found that the same relation is recovered in slow solar wind data, where a Kolmogorov phenomenology is at work, in MHD, a different regime can be found, as described a long time ago [4,31]. In fact, in MHD, when the magnetic effects are dominant, the nonlinear interactions are slowed down by the sweeping effect of fluctuations on the background magnetic field, namely nonlinear interactions happen between fluctuations propagating in the opposite direction with respect to the magnetic field, and the sweeping acts to modify the interaction time. This gives rise to the Kraichnan phenomenology of MHD turbulence [1], which predicts a scaling for Elsässer fluctuations $\delta z^{\pm }∼\ell^{1/2}$ (Elsässer variables are defined as $z^{\pm }=v\pm B/\sqrt{4\pi \rho }$, where v is the plasma velocity, B is the magnetic field, and ρ is the plasma density), thus recovering the Kraichnan spectrum for pseudo-energies $E^{\pm }\left(k\right)∼k^{-3/2}$.

**Figure 7:**
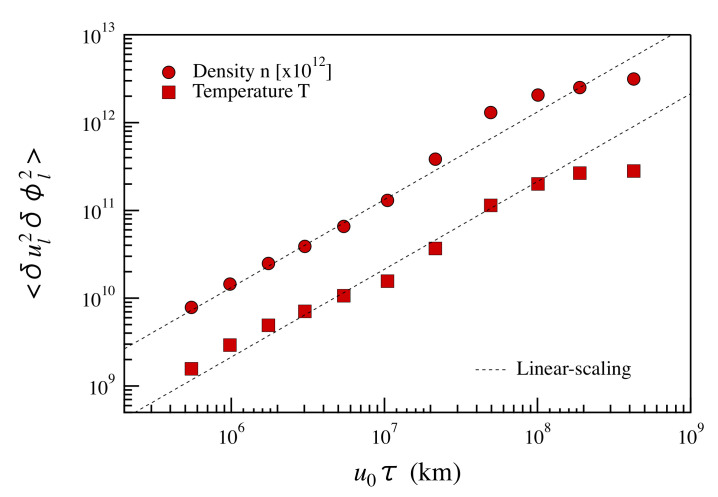
The mixed fourth-order structure functions for the proton density (red circles) and the proton temperature (red squares) as a function of $u_{0}\tau$ for the fast solar wind period. Dashed lines correspond to linear scaling. The density mixed fourth-order structure function was scaled by a factor $\times 10^{12}$.

As an order of magnitude estimate, we can interpret in the same framework the passive scalar fluctuations δϕ, namely the energy transfer rate at a scale *ℓ* is proportional to $\epsilon_{\phi }∼\delta \phi_{\ell }^{2}/t_{\ell }$, $t_{\ell }$ being the effective time needed for an efficient cascading process. Introducing the Alfvén time needed to decorrelate swept fluctuations at the scale *ℓ* with the Alfvén speed, say $t_{A}∼\ell /c_{A}$, we get $t_{\ell }∼\tau_{\ell }^{2}/t_{A}$, where $t_{\ell }∼\ell /\delta u$ is the usual eddy-turnover time. In this case, $\epsilon_{\phi }∼\delta u_{\ell }^{2}\delta \phi_{\ell }^{2}/\ell c_{A}$. By using these phenomenological arguments and by assuming no scaling for $\epsilon_{\phi }$, we obtain:

(12) $$<\delta u_{\ell }^{2}\delta \phi_{\ell }^{2}>∼<\epsilon_{\phi }>c_{A}\ell$$

as an alternative to the usual mixed third-order regular scaling. The scaling of the fourth-order mixed moment can be tested on both density and temperature. Figure 7 shows the obtained results for the mixed four-order structure functions of proton density and temperature in the case of the fast solar wind interval. A linear scaling covering two decades is recovered in this case, suggesting that the above reasoning, although empirical, can be physically grounded.

At the present moment, we show that the linear scaling relation for the fourth-order mixed moment could be interpreted in terms of the different phenomenology of turbulent cascade for MHD with high cross-helicity, as introduced by Kraichnan [4]. We remark that our results are obtained assuming that the anisotropy of the fluctuations does not have a great effect on the scaling features. However, this is the starting point for a theoretical development of the behavior of passive scalar turbulence in the framework of MHD theory. This will also include more investigations to correctly characterize the physics behind the dissipation rates for passive scalars that could be linked to the role of thermal processes in the energy transfer across the inertial cascade. Moreover, deeper investigations are needed to characterize the link between the mixed fourth-order structure function and the Kraichnan phenomenology of MHD turbulence, as well as the role of field anisotropy in changing our results. This topic will be the central issues of future works.

## Figures and Tables

**Table 1:** Observed scaling features.

Quantity	Period	Fit Range [$10^{6}$ km]	*A*	α
Density $n_{p}$	Fast SW	[0.27; 25.0]	$∼10^{-8}$	[0.85±0.03]
Density $n_{p}$	Slow SW	[0.55; 50.0]	$∼10^{-8}$	[1.02±0.05]
Temperature $T_{p}$	Fast SW	[0.27; 25.0]	$∼3\times 10^{4}$	[0.65±0.02]
Temperature $T_{p}$	Slow SW	[0.55; 50.0]	∼160	[0.95±0.05]

## Abbreviations

The following abbreviations are used in this manuscript:

A.U.	Astronomical Unit
DAMF	Dominant Amplitude Multifractal Formalism
EMD	Empirical Mode Decomposition
HGI	Heliographic Inertial Coordinate System
IMF	Intrinsic Mode Functions
KOC	Kolmogorov–Obukhov-Corrsin
Lat	Latitude
MHD	Magnetohydrodynamics
SW	Solar Wind
SWOOPS	Solar Wind Observations Over the Poles of the Sun
UT	Universal Time
